# Metabolic memory in diabetic foot syndrome (DFS): MICRO-RNAS, single nucleotide polymorphisms (SNPs) frequency and their relationship with indices of endothelial function and adipo-inflammatory dysfunction

**DOI:** 10.1186/s12933-023-01880-x

**Published:** 2023-06-26

**Authors:** Alessandro Del Cuore, Rosaria Maria Pipitone, Alessandra Casuccio, Marco Maria Mazzola, Maria Grazia Puleo, Gaetano Pacinella, Renata Riolo, Carlo Maida, Tiziana Di Chiara, Domenico Di Raimondo, Rossella Zito, Giulia Lupo, Luisa Agnello, Gabriele Di Maria, Marcello Ciaccio, Stefania Grimaudo, Antonino Tuttolomondo

**Affiliations:** 1 Department of Promoting Health, Maternal-Infant, Excellence and Internal and Specialized Medicine (PROMISE) G. D’Alessandro, University of Palermo, Piazza Delle Cliniche N.2, 90127 Palermo, Italy; 2 Internal Medicine and Stroke Care Ward, Policlinico “P. Giaccone”, Palermo, Italy; 3 Institute of Clinical Biochemistry, Clinical Molecular Medicine and Laboratory Medicine, Department of Biomedicine, Neurosciences, and Advanced Diagnostics, University of Palermo, Palermo, Italy

**Keywords:** Metabolic memory, Diabetic foot, Epigenetics, SNPs, microRNA

## Abstract

**Background:**

Diabetic foot is a significant cause of morbidity in diabetic patients, with a rate that is approximately twice that of patients without foot ulcers. “Metabolic memory” represents the epigenetic changes induced by chronic hyperglycaemia, despite the correction of the glucose levels themselves. These epigenetic modifications appear to perpetuate the damage caused by persistently elevated glucose levels even in their absence, acting at various levels, mostly affecting the molecular processes of diabetic ulcer healing.

**Methods:**

The aim of our cross-sectional study was to analyse a cohort of patients with diabetes with and without lower limb ulcers.

We examined the effects of epigenetic changes on miRNA 126, 305, and 217 expression and the frequency of the SNPs of genes encoding inflammatory molecules (e.g., IL-6 and TNF-alpha) and their correlations with serum levels of proangiogenic molecules (e.g., ENOS, VEGF and HIF-1alpha) and several adipokines as well as with endothelial dysfunction, assessed noninvasively by reactive hyperaemia peripheral artery tonometry.

Between March 2021 and June 2022, 110 patients were enrolled into the study: 50 diabetic patients with diabetic foot injuries, 40 diabetic patients without ulcerative complications and 20 nondiabetic patients as the control group.

**Results:**

Diabetic subjects with lower limb ulcerative lesions exhibited higher levels of inflammatory cytokines, such as VEGF (191.40 ± 200 pg/mL vs. 98.27 ± 56.92 pg/mL vs. 71.01 ± 52.96 pg/mL; p = 0.22), HIF-1alpha (40.18 ± 10.80 ng/mL vs. 33.50 ± 6.16 ng/mL vs. 33.85 ± 6.84 ng/mL; p = 0.10), and Gremlin-1 (1.72 ± 0.512 ng/mL vs. 1.31 ± 0.21 ng/mL vs. 1.11 ± 0.19 ng/mL; p < 0.0005), than those without lower limb ulcers and healthy controls. Furthermore, we observed that miR-217-5p and miR-503-5p were 2.19-fold (p < 0.05) and 6.21-fold (p = 0.001) more highly expressed in diabetic foot patients than in healthy controls, respectively. Additionally, diabetic patients without lower limb ulcerative complications showed 2.41-fold (p = 0) and 2.24-fold (p = 0.029) higher expression of miR-217-5p and miR-503-5p, respectively, than healthy controls. Finally, diabetic patients with and without ulcerative complications of the lower limbs showed higher expression of the VEGFC2578A CC polymorphism (p = 0.001) and lower expression of the VEGFC2578A AC polymorphism (p < 0.005) than the healthy control population. We observed a significant increase in Gremlin-1 levels in patients with diabetic foot, suggesting that this inflammatory adipokine may serve as a predictive marker for the diagnosis of diabetic foot.

**Conclusions:**

Our results highlighted that patients with diabetic foot showed predominant expression of the VEGF C2578A CC polymorphism and reduced expression of the AC allele. Additionally, we found an overexpression of miR-217-5p and miR-503-5p in diabetic patients with and without diabetic foot syndrome compared with healthy controls.

These results align with those reported in the literature, in which the overexpression of miR-217-5p and miR-503-5p in the context of diabetic foot is reported. The identification of these epigenetic modifications could therefore be helpful in the early diagnosis of diabetic foot and the treatment of risk factors. However, further studies are necessary to confirm this hypothesis.

**Supplementary Information:**

The online version contains supplementary material available at 10.1186/s12933-023-01880-x.

## Background

Diabetic foot is a significant cause of morbidity in diabetic patients, with a rate that is approximately twice that of patients without foot ulcers [1–3].

There has been much debate in the literature about the role of genetics, particularly epigenetic modifications, in the pathogenesis of one of the most costly and severe complications of diabetes mellitus, namely, diabetic foot syndrome (DFS).

Despite the correction of glycaemia, chronic elevation of glucose levels leads to epigenetic changes in some diabetic patients, known as "metabolic memory", and these changes result in the formation of classic vascular complications, including "diabetic foot" [1–6].

"Metabolic memory" perpetuates its damage by slowing the ulcer healing process and by promoting susceptibility to infections in patients with diabetes.

The epigenetic modifications induced by "metabolic memory" affect various molecular targets, such as miRNAs and single nucleotide polymorphisms of some genes encoding proteins implicated in the four stages of diabetic ulcer healing [5, 6].

Over the years, several miRNAs involved in epigenetic changes in diabetic ulcers have been studied. Among these, the depletion of miRNA 126, driven by and correlated with the concomitant overexpression of miRNA 503 induced by chronically elevated glucose values, seems to play a relevant role [5].

Another miRNA, miRNA 217, has also been studied since it is involved in the pathogenesis of diabetic ulcers and is related to the hypoxia-inducible factor (HIF-1alpha) pathway [7].

Dhamodharan and Viswanathan showed that the rs1800629 -G308A SNP of the TNF-α gene conferred genetic susceptibility to both conditions [8, 9].

Erdogan et al. showed that the IL-6 -174 G > C gene polymorphism may represent a potential genetic marker, as its presence appears to predict individual susceptibility to the onset of diabetes mellitus but not to the development diabetic foot [10].

The role of genetic polymorphisms of molecules involved in angiogenesis, such as VEGF and ENOS, has been much debated in the context of diabetic foot ulcer damage [11–14]. Corapcioglu et al. in 2010 studied the association of the SNP rs1799983 of the ENOS-G894T gene, and they reported that this polymorphism was not associated with a diabetic ulcer or further diabetes-related complications, except in the presence of atherosclerotic cardiovascular disease [11]. Xiaolei Li et al. found a reduced trend in the expression of the A allele of the rs699947 -C2578A SNP of the VEGF gene in patients with DFS compared to controls, suggesting a correlation of this polymorphism with a reduced risk of developing diabetic foot [12]. In a 2011 paper, Amoli et al. highlighted that the frequency of the AA 2578 single nucleotide polymorphism of the VEGF gene was significantly reduced in patients with DFS [13].

Pichu et al. highlighted the role of hypoxia-inducible factor 1 alpha (HIF-1alpha) in the pathogenesis of diabetic ulcers. Specifically, an association was found between the SNP rs11549465 of the HIF-1alpha C1772T exon 12 gene and the SNP rs11549467 of the HIF-1alpha G1790A exon 12 gene and susceptibility to developing diabetic foot ulcers, as well as the presence of reduced serum levels of HIF-1alpha in patients with DFS [15, 16].

Gremlin-1 is an inflammatory adipokine produced by adipose, muscle, and liver cells that can oppose the effects of insulin, and it represents a novel marker of adipose inflammation [17].

Currently, surrogate indices of cardiovascular disease, such as endothelial function indices and serum levels of inflammatory adipokines, in a diabetic population with ulcers of the lower extremities have been evaluated in only a few studies [1–3].

To the best of our knowledge, there are no studies in the literature reporting a correlation of the effects of epigenetic changes on the expression of miRNAs 126, 305, and 217 and on the frequency of SNPs of genes encoding inflammatory molecules, such as IL-6 and TNF-alpha, and proangiogenic molecules, such as ENOS, VEGF and HIF-1alpha, with endothelial dysfunction, assessed noninvasively by RH-PAT, or with serum levels of inflammatory molecules and adipokines in patients with diabetes with and without lower limb ulcers.

Based on these premises, the aim of our study was to evaluate whether diabetic patients with foot ulcers (due to DFS), compared to a population with diabetes without DFS and healthy controls, present effects of epigenetic changes on the expression of miRNAs and to evaluate the frequency SNPs of genes encoding inflammatory and proangiogenic molecules to highlight a possible genetic predisposition to developing foot ulcers in these patients.

Other aims were therefore to identify a possible molecular therapeutic target in the pathogenesis of diabetic foot and to show whether these alterations may be associated with a more extensive inflammatory pathway and a greater degree of endothelial dysfunction.

### Aims of the study

1. The main objective of our study is to assess, in a population of diabetic patients with and without foot complications compared to a population of healthy control subjects, the effects of epigenetic changes on the expression of miRNAs 126, 503 and 217 and on the frequency of the following polymorphisms:
2. *SNP rs1800795 del gene IL-6 -G174C.*
3. *SNP rs1800629 del gene TNF-α -G308A.*
4. *SNP rs699947 del gene VEGF-C2578A.*
5. *SNP rs3025039 del gene VEGF C936T.*
6. *SNP rs1799983 del gene ENOS-G894T.*
7. *SNP rs11549465 del gene HIF-1alpha C1772T.*
8. A further objective was to assess some possible relationship between the effects of these epigenetic changes and serum levels of inflammatory markers (IL-6; TNF-alpha, ENOS, VEGF, and HIF-1alpha), serum levels of inflammatory adipokines (Gremlin-1) and indices of endothelial dysfunction in the patient population under study.

## Materials and methods

Between March 2021 and June 2022, we enrolled all patients suffering from diabetic ulcerative lesions of the lower limbs hospitalized in our Internal Medicine and Stroke Care Ward of the Policlinico “P. Giaccone” Hospital of the University of Palermo.

The WHO defines diabetic foot syndrome (DFS) as the presence of an ulcerative lesion of the foot (including the ankle) related to neuropathy and varying degrees of ischaemia and infection [18]. Therefore, an ulcerative foot injury is defined as a continuous skin injury requiring more than 14 days for healing [19].

During this period, we also recruited as patients with diabetes without foot ulcers who were hospitalized in our department for clinical complications related to diabetes (e.g., glycaemic imbalance, hypoglycaemia, and skin lesions) and a population of healthy controls without diabetes who were hospitalized in our division for other causes.

All patients gave informed consent to participate in the study and to disclose the data according to the principles of the Declaration of Helsinki (2001).

All patients with inflammatory or infectious diseases, autoimmune or rheumatic diseases, neoplasms, haematological diseases, severe kidney or liver failure, fever, and recent hospitalization within the last month, as well as those receiving treatment with anti-inflammatory drugs, were excluded from the study.

All subjects with diabetic foot syndrome were matched for age (± 3 years) and sex with diabetic subjects without foot ulcers and with healthy subjects.

Diabetic peripheral neuropathy was assessed by collecting the patient's anamnestic information and through clinical evaluation and diagnostic tests.

The Neuropathy Symptom Score (NSS) [20], which is generally used in clinical practice since it shows high validity and sensitivity, was used to assess neuropathy symptoms. In addition, the Semmes‒Weinstein monofilament test was used to estimate diabetic peripheral neuropathy [21]. Finally, a careful, objective examination of the lower limbs was carried out to look for the presence of the following features: hammer or claw foot, Charcot deformity, hallux limitus, prominent metatarsal heads, hallux valgus, and bony prominences. In addition, the ankle-heel angle was measured with a goniometer.

The diagnosis of type 2 diabetes mellitus was based on the revised criteria of the American Diabetes Association (ADA), using random blood glucose values > 126 mg/dl or using a clinical algorithm that took into account the age of onset of the disease, the present symptoms and weight, the family history, the start of insulin treatment, and the history of ketoacidosis [22].

Hypertension was defined using the 2018 ESC criteria [23].

Dyslipidaemia was defined by triglyceride levels ≥ 150 mg/dl and HDL-cholesterol levels < 40 mg/dl related to the patient's sex [24].

### Laboratory analysis

Clinical and anthropometric data were collected at the time of enrolment. Patients were classified as obese (BMI ≥ 30), overweight (BMI 25–29.9), and normal weight (BMI 18.5–24.9 kg/m2).

At the time of enrolment, blood samples were collected to assess ALT, triglyceride, blood glucose, total cholesterol and HDL-cholesterol levels.

### Single nucleotide polymorphism analysis

Genetic analysis was performed as follows:

After obtaining informed consent from patients, EDTA-collected peripheral blood or plasma and serum samples were collected and stored at − 20 °C until DNA extraction.

DNA was extracted on a column using affinity chromatography with the QiaAMP DNA Blood Mini Kit (Qiagen). DNA yield and integrity were assessed by measuring optical density using a UV spectrophotometer (ratio 260/280 nm < 2).

Single nucleotide polymorphisms (SNPs) were evaluated through real-time PCR using an allelic discrimination assay with allele-specific TaqMan probes containing the fluorochromes FAM or VIC as markers.

The following assays were used:

- SNP rs1800795 of the IL-6 gene -174, G > C.
- SNP rs1800629 of the TNF-α-308 gene, G > A.
- SNP rs699947 of the VEGF-2578 gene, C > A.
- SNP rs11549465 of the HIF-1alpha-1772 gene, C > T.
- SNP of the ENOS-894 gene, G > T.

Amplification was performed using the StepOnePlus Real-Time PCR System supplied by Applied Biosystems, and the results were analysed using SDS software, version 2.3. The data were collated into a diagram, which reported the fluorescence associated with each allelic variant related to each SNP studied.

Each allelic discrimination run was validated by the use of a homozygous sample for allele 1, a homozygous sample for allele 2 and a heterozygous sample for the polymorphisms in question, previously determined by another method (sequencing). In addition, at least three NTCs (no template controls) were randomly inserted into the plate containing all the reagents except the DNA, replaced by DEPC water, to highlight any contamination that would have invalidated the assay. Regarding result interpretation, the response is unambiguous and defines the homozygote status for one or the other allele or heterozygote status of the subject.

### miRNA analysis (RNA Extraction, real-time PCR and statistical analysis)

Total RNA was purified from the serum samples of 30 patients using the commercially available miRNeasy Serum/Plasma Kit (cat. Number 217204; Qiagen) and miRCURY LNA RNA Spike-In kit (cat. Number 339390; Qiagen) according to the manufacturer’s instructions. For miRNA expression, 1 μg of RNA was reverse transcribed to cDNA using the miRCURY LNA kit (cat. Number 339340; QIAGEN) according to the manufacturer’s recommendations. Quantitative real-time PCR was performed using the miRCURY LNA SYBR Green PCR kit (cat. Number 339346; Qiagen) and the Custom_miRCURY PCR PCR Panel (96-well format, Cat. No. 339330, YCA41200, Qiagen). The plates contained primers to analyse 3 targets, hsa-miR-217-5p, hsa-miR126-3p and hsa-miR-503-5p3, and 4 housekeeping genes, hsa-miR-93-5p, hsa-miR-191-5p, hsa-miR-425-5p and hsa-miR-451a, as reported in Additional file 1: Table S1. In addition, each plate contained 4 UniSp RNA spike-ins: UniSp2 and UniSp4 (RNA isolation controls), UniSp3 (interplate calibrator assay) and UniSp6 (cDNA synthesis control), as reported in Additional file 1: Table S1. The mean threshold cycle was used for the calculation of the relative expression using hsa-miR-451a as the reference gene. Data are expressed as the fold change using the 2 − ΔΔCt method with the healthy group or diabetic patients without foot ulcers as the control group. Differences among experimental groups were analysed by Student’s t test. Student’s t test for independent experiments was performed to test differences in the fold expression of genes between the experimental groups; the Bonferroni correction for multiple hypothesis testing was applied to the t test results; and the P value of the false-positive error probability was used as the primary criterion for the selection of genes (P value cut-off of 0.05 for statistical significance). Then, the fold change was considered a measure of biological significance. The cycle threshold (ct) values were submitted to Web-based PCR Array Data Analysis software (https://geneglobe.qiagen.com/it/analyze. For all experiments, the data are represented as the mean ± S.D. The statistical significance of the differences between a single group and the relative control was evaluated by a two-tailed Student’s t test. All statistical analyses were performed using R Statistical Software version 4.0.4 (Core 236 Team, 2021).

### Biochemical analysis

Biochemical analysis was performed as follows:

Serum levels of Gremlin-1, TNF-alpha, VEGF, HIF-1alpha, and IL-6 were assessed in blood samples. Serum was obtained by centrifugation at 3000 rpm for 10 min and stored at a temperature of − 80 °C. All models were analysed in the same analytical session. The serum concentrations of Gremlin-1 (Biomatik), TNFalpha, VEGF, HIF-1alpha, IL-6, and eNOS (Thermo Fisher) were measured using the respective ELISA kits.

### Evaluation of cognitive performance

The Mini-Mental State Examination (MMSE), an 11-question questionnaire, was administered to assess the five primary cognitive areas (language, attention, orientation, memorization, and repetition and calculation). The maximum score is 30. MMSE scores below 24 (23 or lower) suggest mild cognitive impairment [25].

### Evaluation of endothelial indices

The RH-PAT principle has been described previously in several studies [4]. Briefly, a blood pressure cuff was placed on an upper limb, while the contralateral arm was used as a control. The PAT probe was placed on the finger of each of the two hands. After a 5-min control measurement period, the blood pressure cuff was inflated to 60 mmHg above the previously measured systolic pressure or up to 200 mmHg for 5 min and then deflated to induce reactive hyperaemia. The RH-PAT data were digitally analysed using Endo-PAT2000 device software, version 3.0.4. The RH-PAT index reflected the extent of reactive hyperaemia. The RH-PAT index was calculated as the ratio of the average of the PAT signal amplitudes over the first minute of initial measurement, beginning 1.5 min following pressure cuff deflation (A: Control Arm; C: Occluded Arm), divided by the average of the PAT signal amplitudes over 2.5 min before pressure cuff inflation (B: Control Arm; D: Occluded Arm). This RH-PAT index, called the RHI (reactive hyperaemia index), is expressed by the formula RHI = (C/D)/(A/B) x basal correction. RHI values < 1.67 indicate endothelial dysfunction.

### Statistical analysis

The sample size was estimated to detect a 25% mean difference in clinical variables between patients groups with a SD of 0.5. The sample size of 17 patients for each of the 2 groups was calculated to provide 80% power with α = 0.05. The univariate analysis of variance (ANOVA) was performed for parametric variables, and post hoc analysis with the Tukey test was used to determine whether there were pairwise intragroup differences (see red font on “Statistical analysis” section.

Quantitative and qualitative statistical data analysis was performed for all items, including descriptive statistics. Continuous variables are expressed as the mean values ± standard deviations (SD) unless otherwise specified. Fundamental significant differences between the groups were assessed using the chi-square test or Fisher's exact test for categorical variables and by univariate analysis of variance (ANOVA) for parametric variables. Multivariable logistic regression analysis was used to examine the correlations between patient characteristics (independent variables) and subgroups (dependent variables) in a multiple regression model. In addition, odds ratios (ORs) and their 95% confidence intervals (CIs) were calculated and adjusted for drug therapy as a covariate. Finally, Pearson's correlation analysis was conducted to assess the association between the indices of endothelial dysfunction and other clinical, laboratory, and instrumental variants in the various groups. The data were analysed using SPSS Software, version 22.0 (IBM Corp., Armonk, NY, USA). P values less than 0.05 were considered statistically significant.

## Results

Between March 2021 and June 2022, a cohort of 50 diabetic patients with diabetic foot injuries hospitalized admitted to our Ward of Internal Medicine with Stroke Care of the Policlinico ‘Paolo Giaccone’ University Hospital of Palermo was enrolled and compared with a control group of 40 diabetic patients without ulcerative complications and with a population of 20 nondiabetic patients also hospitalized in our ward for nondiabetes-related problems and other causes.

The laboratory, demographic and general variables of the diabetic patients with and without ulcerative foot lesions, as well as of the healthy control subjects, are presented in Table 1.

**Table 1:** Demographic and clinical variables in Diabetes Mellitus type 2 patients with and without Diabetic Foot Syndrome and in healthy controls

Variables	DFS (n = 50)	NDFS (n = 40)	Controls (n = 20)	p
Sex M/F	37/13	27/13	12/8	0.524
Hypertension (n/%)	38/76	29/70	8/40	0.014
Cardiovascular events (n/%)	18/36	12/22	4/20	0.407
Stroke (n/%)	5/10	12/22	2/10	0.407
Dyslipidemia (n/%)	36/72	18/38	4/20	< 0.0005
Smoking (n/%)	24/48	9/23	9/45	0.008
Beta-blockade (n/%)	19/38	16/40	5/25	0.529
Calcium channel blockers (n/%)	14/28	7/18	5/25	0.555
ACE inhibitors or ARB (n/%)	23/46	17/40	3/15	0.052
Statin (n/%)	34/68	19/50	5/25	0.004
Antiplates drugs (n/%)	34/68	14/35	6/30	0.001
GLP-1-ra or a DPP-4 inh (n/%)	5/10	19/48	0/0	0.000
Sulfonylurea (n/%)	2/4	5/13	0/0	0,103
Metformin (n/%)	9/18	22/50	1/5	< 0.0005
Insulin therapy (n/%)	41/82	13/35	0/0	< 0.0005

M/F: males/females; ACE: angiotensin converting enzyme; GLP1-ra: GLP-1 receptor agonist; DPP-4 inh: DPP-4 inh: dipeptidyl peptidase-4 inhibitors;

The univariate analysis (Table 2) showed that patients with diabetic foot syndrome had lower RHI values than healthy controls (1.42 ± 0.49 vs. 2.03 ± 0.66; p < 0.005) and RHI values that were comparable to those of the diabetic patients without ulcerative complications (1.42 ± 0.49 vs. 1.67 ± 0.43).

**Table 2:** Results of one-way analysis of variance for diabetes mellitus type 2 patients with and without diabetic foot syndrome and for controls

Variables	DFS (n = 50)	NDFS (n = 30)	Controls (n = 30)	p
Age (years) (mean ± SD)	68,08 ± 9.81	66.95 ± 11,92	67.05 ± 13,82	0.881
SBP (mmHg) (mean ± SD)	130.34 ± 17.52	129.37 ± 17.25	125.00 ± 12.24	0.47
DBP (mmHg) (mean ± SD)	72.22 ± 10.40	74.85 ± 14,64	74.500 ± 10,85	0.59
Height (cm) (mean ± SD)	167.96 ± 15,19	169.90 ± 7,71	164.20 ± 7,82	0.21
Weight (kg) (mean ± SD)	82.98 ± 19.87	83.80 ± 22.19	72.75 ± 13.16	0.95
BMI (kg/m^2^) (mean ± SD)	28.58 ± 6.32	28,98 ± 6.54	27.11 ± 5.61	0.54
RHI (mean ± SD)	1.42 ± 0.49	1.67 ± 0.43	2.03 ± 0.66	** < 0.0005**
MMSE (mean ± SD)	25.62 ± 3.36	26.35 ± 3.53	29,00 ± 1.29	** < 0.0005**
HbA_1c_ (%) (mean ± SD)	8.07 ± 1.46	7.68 ± 2.02	5,89 ± 0.57	0.32
IL-6 (pg/mL) (mean ± SD)	25.99 ± 52.73	5.04 ± 4.92	3,09 ± 1.91	0.70
TNF-alpha (pg/mL)(mean ± SD)	3,.46 ± 3.08	2.97 ± 0.71	2.15 ± 0.90	0.304
VEGF (pg/mL)(mean ± SD)	191.40 ± 200	98,27 ± 56.92	71.01 ± 52.59	**0.022**
eNOS (ng/mL)(mean ± SD)	2.47 ± 8.19	1.01 ± 1.67	1.063 ± 1.19	0.606
HIF-1alpha (ng/mL)(mean ± SD)	40.18 ± 10.80	33.50 ± 6.16	33.85 ± 6.84	**0.010**
Gremlin-1 (ng/mL)(mean ± SD)	1.72 ± 0.512	1.31 ± 0.21	1.11 ± 0.19	** < 0.0005**

SBP: systolic blood pressure; DBP: diastolic blood pressure; BMI: body mass index; RHI: reactive hyperaemia index; MMSE: mini-mental state examination; HbA_1c:_ glycated haemoglobin; TNF-alfa: tumor necrosis factor-alfa; VEGF: Vascular Endothelial Growth Factor; NOS: endothelial Nitric-Oxid Synthase; HIF-1 alfa: *Hypoxia-inducible factor 1*-*alpha*

Patients with DFS had a higher frequency of hypertension (76% vs 70%) and dyslipidaemia (72% vs 38%), and a higher use of Ace inhibitors/Angiotensin receptor blockers (ARBs) (46% vs 15%) and Statins (68% vs 50%) compared to healthy controls.

Patients with diabetic foot syndrome exhibited a higher percentage of smoking habits (48% vs 22.50%) compared to diabetic controls alone.

Moreover, the cohort of diabetic patients without lower limb ulcer complications had a higher use of metformin (50% vs 18%) and GLP-1 analogues/anti DPP4 (48% % vs 10% vs 0.57%) compared to diabetic foot patients and healthy controls.

The cohort of patients with diabetic ulcers showed lower scores on the age-adjusted MMSE than healthy controls (25.62 ± 3.36 vs. 28.7 ± 1.29; p < 0.005) and MMSE scores that were comparable to those of diabetic controls (25.62 ± 3.36 vs. 26.35 ± 3.53).

Diabetic subjects with lower limb ulcerative lesions exhibited higher levels of inflammatory cytokines than patients with diabetes without lower limb ulcerative lesions and healthy controls, such as VEGF (191.40 ± 200 pg/mL vs. 98.27 ± 56.92 pg/mL vs. 71.01 ± 52.96 pg/mL p = 0.22), HIF-1alpha (40.18 ± 10.80 ng/mL vs. 33.50 ± 6.16 ng/mL vs. 33.85 ± 6.84 ng/mL p = 0, 10), and Gremlin-1 (1.72 ± 0.512 ng/mL vs. 1.31 ± 0.21 ng/mL vs. 1.11 ± 0.19 ng/mL p < 0.0005), and comparable levels of IL-6 (25.99 ± 52.73 pg/mL vs. 5.04 ± 4.92 pg/mL vs. 3.096 ± 1, 91 pg/mL p = 0.70), TNF-alpha (3.46 ± 3.08 pg/mL vs. 2.97 ± 0.71 pg/mL vs. 2.15 ± 0.91 pg/mL p = 0.304), and eNOS (2.47 ± 8.19 ng/mL vs. 1.01 ± 1.67 ng/mL vs 1.06 ± 1.19 ng/mL p = 1.00).

At Multi-variable logistic regression analysis (Table 3) a MMSE score < 24 was significantly correlated to hypertension (B:2.66; p = 0.027) and a previous stroke (B:2.58; p = 0.040), whereas no significant association have been observed between a RHI < 1.6 and other clinical and laboratory variables (Table 3).

**Table 3:** logistic regression analysis of clinical and laboratory variables associated to a MMSE < 24 and to a RHI > 1.6 in subjects with diabetic foot

Parameter estimates								
MMSE SCORE^a^	B	Std. Error	Wald	df	P	Exp(B)	95% Confidence Interval for Exp(B)	
							Lower bound	Upper bound
Intercept	1.317	5.034	0.068	1	0.794			
Sex	0.302	0.908	0.111	1	0.739	1.353	0.228	8.025
Age	0.095	0.054	3.041	1	0.081	1.099	0.988	1.223
SBP	− 0.063	0.038	2.655	1	0.103	0.939	0.871	1.013
DBP	0.021	0.039	0.300	1	0.584	1.022	0.946	1.103
BMI	− 0.082	0.065	1.589	1	0.208	0.922	0.812	1.046
Hypertension	2.666	1.208	4.873	1	0.027	14.386	1.348	153.499
CV Events	− 0.116	0.887	0.017	1	0.896	0.890	0.157	5.063
Stroke	2.582	1.259	4.202	1	0.040	13.218	1.120	156.029
Dyslipidemia	1.795	0.998	3.234	1	0.072	6.021	0.851	42.603
HbA_1c:_	0.012	0.241	0.003	1	0.959	1.012	0.632	1.622
Smoke	0.395	0.793	0.248	1	0.618	1.484	0.314	7.023
Beta-blockers	− 1.282	0.951	1.814	1	0.178	0.278	0.043	1.792
Calcium antagonist	− 0.842	1.000	0.710	1	0.400	.431	0.061	3.057
ACE I/ ARBs	− 0.544	0.854	0.405	1	0.524	.581	0.109	3.098
Statins	− 0.111	0.857	0.017	1	0.897	o.895	o.167	4.805
Antiplatelets	− 0.038	0.985	0..002	1	0.969	0.962	0.140	6.635
GLP1 A/Anti-DPP4	− 0.625	1.113	0.316	1	0.574	0.535	0.060	4.737
Sulfonylureas	− 1.331	1.727	0.594	1	0.441	0.264	0.009	7.798
Metformin	− 3.469	1.674	4.294	1	0.038	0.031	0.001	0.829
Insulin	0.086	1.090	0.006	1	0.937	1.089	00.129	9.229
RHI								

Parameter estimates									
RHISCORE^a^		B	Std. Error	Wald	df	Sig	Exp(B)	95% Confidence Interval for Exp(B)	
								Lower bound	Upper bound
0	Intercept	− 3.277	6.112	0.287	1	0.592			
	Sex	− 0.014	1.114	0.000	1	0.990	0.986	0.111	8.759
	Age	0.0003	0.048	0.005	1	0.943	1.003	0.913	1.102
	SBP	0.080	0.048	2.815	1	0.093	1.083	0.987	1.190
	DBP	− 0.035	0.049	0.519	1	0.471	0.966	0.878	1.062
	BMI	− 0.009	0.073	0.016	1	0.900	0.991	0.859	1.143
	Ipertension	− 0.216	0.140	2.387	1	0.122	0.806	0.612	1.060
	CV Events	1.112	1.161	0.918	1	0.338	0. 329	0.034	3.202
	Stroke	1.089	1.146	0.904	1	0.342	2.973	0.315	28.067
	Dyslipidemia	1.394	1.557	0.802	1	0.371	0.248	0.012	5.249
	HbA_1c_:	1.150	0.926	1.543	1	0.214	0.317	0.052	1.944
	Smoke	0.026	0.269	0.009	1	0.922	0.974	0.575	1.651
	Intercept	0.497	0.959	0.269	1	0.604	1.644	0.251	10.763
	Beta-blockers	0.566	0.974	0.338	1	0.561	1.762	0.261	11.883
	Calcium antagonist	0.305	1.103	0.076	1	0.782	1.356	0.156	11.773
	ACE I/ ARBs	− 0.108	1.166	0.009	1	0.926	0.898	0.091	8.827
	Statins	1.139	1.079	1.115	1	0.291	3.124	0.377	25.871
	Antiplatelets	− 1.016	1.200	0.717	1	0.397	0.362	0.034	3.801
	GLP1 A/Anti-DPP4	− 1.006	0.964	1.089	1	0.297	0.366	0.055	2.419
	Sulfonylureas	− 2.289	1.717	1.778	1	0.182	0.101	0.004	2.932
	Metformin	1.220	1.281	0.908	1	0.341	3.389	0.275	41.735
	Insulin	0.183	1.157	0.025	1	0.874	1.201	0.124	11.598

a. The reference category is: control subjects

SBP: systolic blood pressure; DBP: diastolic blood pressure; CV events: cardiovascular events; BMI: body mass index; RHI: reactive hyperaemia index; MMSE: mini-mental state examination; HbA_1c_: glycated haemoglobin; ACE-inh/ARBs: ACE-inhbitors/ angiotensin receptor blockers; GLP1-ra: GLP-1 receptor agonist; DPP-4 inh: DPP-4 inh: dipeptidyl peptidase-4 inhibitors

Diabetic patients with and without ulcerative complications of the lower limb showed a higher expression of the VEGFC2578A CC polymorphism (p = 0.001) and a lower expression of the VEGFC2578A AC polymorphism (p < 0.005) compared to the healthy control population (Table 4).

**Table 4:** Distribution of alleles of selected SNPs in subjects with diabetic foot syndrome (DFS), diabetic subjects without diabetic foot syndrome and control subejcts

SNP	Allelic variant	DFS (n = 50)	NDFS (n = 40)	Control (n = 20)	p
IL—6 n/%	GG	35/70	20/63	10/53	0.317
	GC	14/30	11/34	7/32	0.816
	CC	2/2	1/3	3/16	0.175
TNF-α n/%	GG	44/87	26/81	19/59	0.390
	GA	5/10	6/19	0	0.112
	AG	0	0	1/4	0.196
VEGF 2578A n/%	CC	37/74	19/47.5	1/5	0.001
	AA	10/20	14/35	6/30	0.152
	AC	3/6	7/17.5	13/65	< 0.0005
VEGF C936T n/%	CC	38/77	23/72	13/46	0.645
	CT	11/21	8/25	6/14	0.754
	TT	1/ 2	1/3	1/ 4	1.000
eNOS n/%	GG	24/49	13/41	13/45	0.241
	TT	3/6	2/6	0	0.652
	GT	23/43	17/53	7/21	0.459
HIF-1α n/%	CC	37/70	28/88	16/55	0.340
	CT	13/29	4/13	3/7	0.277
	TT	0	0	1/3	0.196

IL-6: Interleukin-6, TNFA-alfa: Tumor necrosis factor alfa; VEGF: Vascular Endothelial Growth Factor: eNOS: endothelial Nitric-Oxid Synthase; HIF-1 alfa: *Hypoxia-inducible factor 1*-*alpha*

**Fig. 1:**
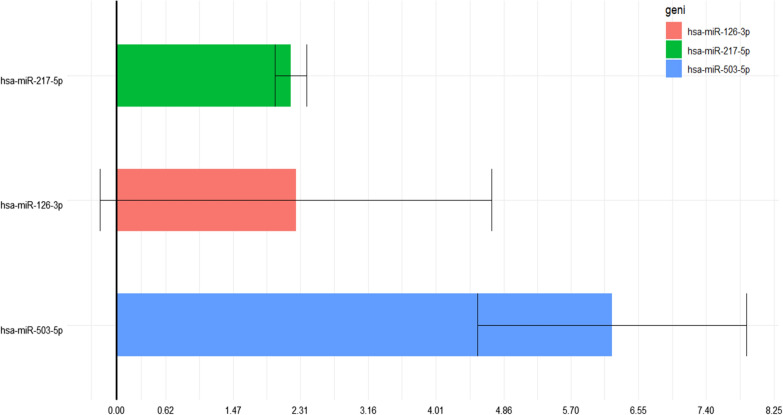
Results of miRNAs expression analysis in the diabetic foot patient group versus control group. hsa-miR-217-5p: human miRNA217-5p; hsa-miR-126-3p: human miRNA126-3p; hsa-miR-503-5p: human miRNA 503-5p

**Fig. 2:**
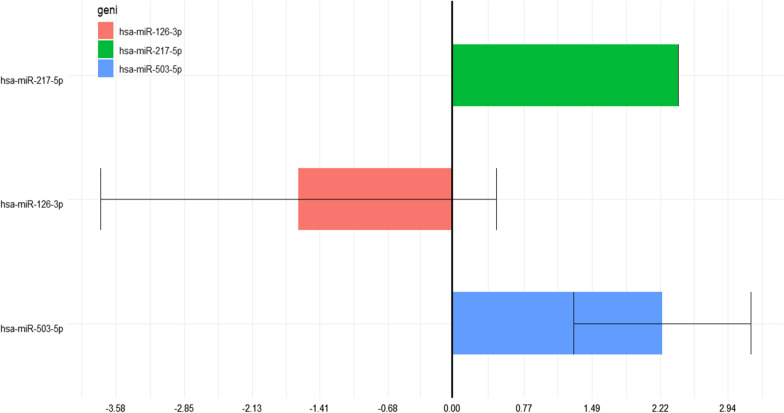
Results of miRNAs expression analysis in the diabetic patient group versus control group. hsa-miR-217-5p: human miRNA217-5p; hsa-miR-126-3p: human miRNA126-3p; hsa-miR-503-5p: human miRNA 503-5p

**Fig. 3:**
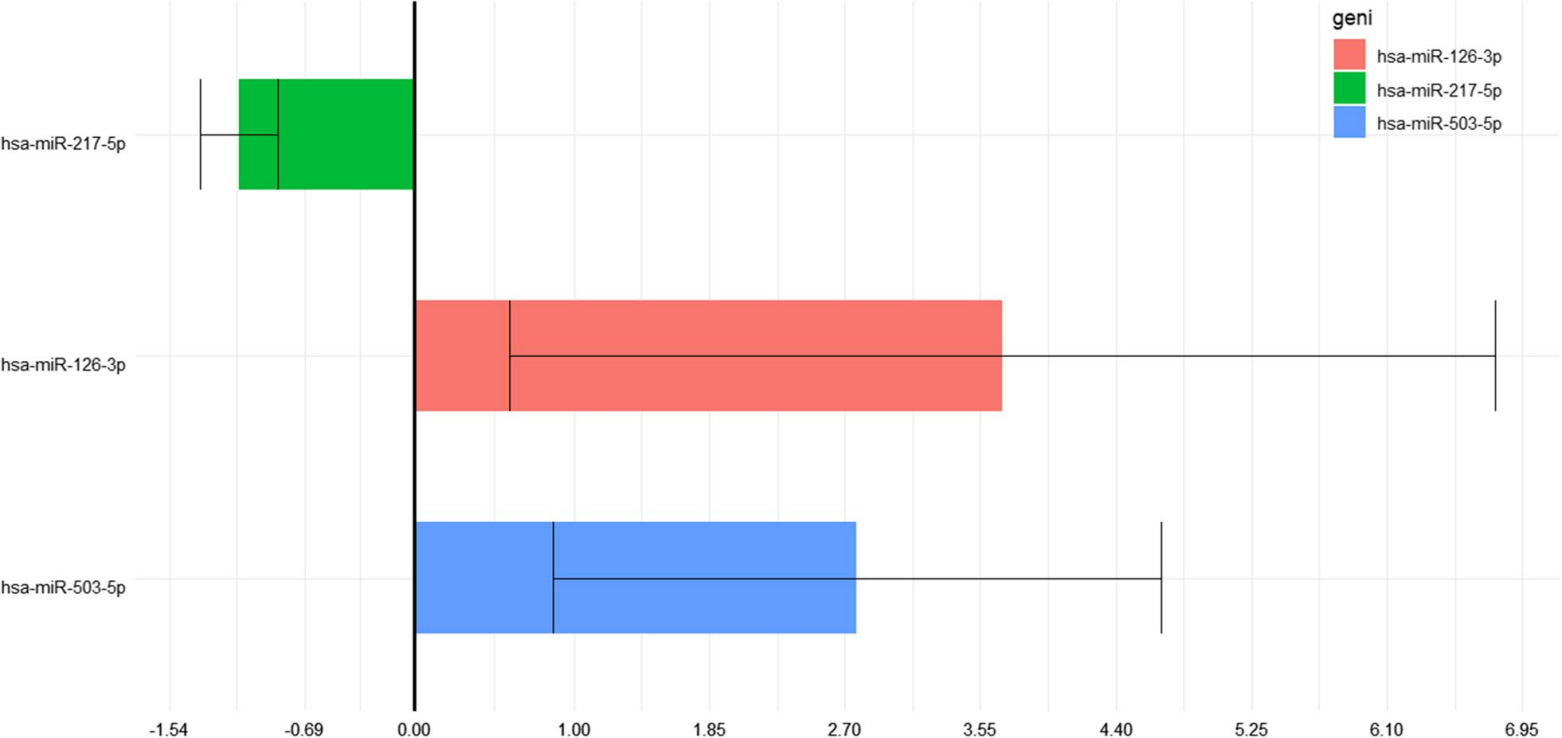
Results of miRNAs expression analysis in the diabetic foot patient group versus diabetic group. hsa-miR-217-5p: human miRNA217-5p; hsa-miR-126-3p: human miRNA126-3p; hsa-miR-503-5p: human miRNA 503-5p

Furthermore, we observed that miR-217-5p and miR-503-5p were respectively 2.19-fold (p < 0.05) and 6.21-fold (p = 0.001) more expressed in diabetic foot patients than in healthy controls (Table 5, Fig. 1). Additionally, diabetic patients without lower limb ulcer complication showed a 2.41-fold and 2.24-fold (p = 0.029) higher expression of miR-217-5p and miR-503-5p, respectively, than healthy controls (Table 6, Fig. 2). Finally, we identified no significant differences in the expression of these miRNAs when comparing diabetic patients with and without the ulcerative lesion of the inferior limb (Table 7, Fig. 3).

**Table 5:** Results of miRNAs expression analysis in the diabetic foot patient group versus control group

Analyses	Mirna	FR	FC	Pval	sd
DFS-CTR	hsa-miR-217-5p	2,190659	2,190659	7,79–11	0,1960859
DFS-CTR	hsa-miR-126-3p	2,256038	2,256038	0,14960987	2,4553849
DFS-CTR	hsa-miR-503-5p	6,218755	6,218755	0,001300872	1,6882705

hsa-miR-217-5p: human miRNA217-5p; hsa-miR-126-3p: human miRNA126-3p; hsa-miR-503-5p: human miRNA 503-5p

**Table 6:** Results of miRNAs expression analysis in the diabetic patient group versus control group

Analyses	Mirna	FR	FC	Pval	sd
DM-CTR	hsa-miR-217-5p	2,413078873000	2,413078873	0	0
DM-CTR	hsa-miR-126-3p	− 1,63456326800	0,6117842	0,290,085,832	2,107731
DM-CTR	hsa-miR-503-5p	2,2414869230000	2,241486923	0,029880933	0,946892

hsa-miR-217-5p: human miRNA217-5p; hsa-miR-126-3p: human miRNA126-3p; hsa-miR-503-5p: human miRNA 503-5p

**Table 7:** Results of miRNAs expression analysis in the diabetic foot patient group versus diabetic group

Analyses	Mirna	FR	FC	PvaI	sd
DFS-DM	hsa-mIR-217-5p	− 1,101531264	0,907827161	0,172758738	0,240155147
DFS-DM	hsa-miR-126-3p	3,687636212	3,687636212	0,161920716	3,093095575
DFS-DM	hsa-miR-503-5p	2,774388046	2,774388046	0,108002414	1,905249426

hsa-miR-217-5p: human miRNA217-5p; hsa-miR-126-3p: human miRNA126-3p; hsa-miR-503-5p: human miRNA 503-5p

At multivariable logistic regression, RHI < 1.6 (B:3,69; p = 0.007), VEGF (B:0.02; p = 0.011) and Gremlin-1 (B:8,75;p = 0,002) serum levels were correlated to DFS in comparison to control subjects. VEGFC2578A CC presence (B:2,55; p = 0,046) and Gremlin-1 (B:5,19; p = 0,039) serum levels were correlated to DFS in comparison to control subjects. A RHI value < 1,6 (B:-2,00; p = 0,019) and HIF-1alpha (B: 0,12; p = 0.024), VEGF (B: -0,01; p = 0,008) and Gremlin-1 (B:3,56; p = 0,010) serum levels were significantly associated to DFS presence in comparison to diabetics without DFS (see Table 8).

**Table 8:** multinomial regression analysis of clinical and laboratory variables associated with diabetes and with DFS (diabetic foot syndrome) in comparison to control healthy subjects and of clinical and laboratory variables associated with diabetic foot con comparison to diabetics without DFS

Parameter Estimates									
		B	Std. Error	Wald	df	Sig	Exp(B)	95% Confidence Interval for Exp(B)	
								Lower bound	Upper bound
1.0	Intercept	− 7.66	4.90	2.44	1	0.118			
	RHI score	− 1.69	1.160	2.12	1	0.145	.184	0.019	1.790
	HIF1-a	0.027	0.08	0.108	1	0.743	1.028	0.873	1.210
	VEGF	0.012	.010	1.48	1	0.223	1.012	0.993	1.032
	GREM-1	5.19	2.52	4.25	1	0.039	180.75	1.295	2522433
	VEGFC 2578ACC	2.55	1.27	3.99	1	0.046	12.868	1.050	157.65
2.0	Intercept	− 18.08	5.71	10.01	1	0.002			
	RHI score	3.69	1.36	7.37	1	0.007	40.34	2.800	581.24
	HIF1-a	0.15	0.09	2.93	1	0.087	1.166	0.978	1.39
	VEGF	0.02	0.01	6.49	1	0.011	1.03	1.007	1.053
	GREM-1	8.75	2.80	9.77	1	0.002	6357.29	26.222	1541301.23
	VEGFC 2578ACC	2.18	1.42	2.35	1	0.125	8.880	0.547	144.12

Parameter Estimates									
GRUPPOSCORE^a^		B	Std. error	Wald	df	Sig	Exp(B)	95% confidence interval for Exp(B)	
								Lower bound	Upper bound
2.0	Intercept	− 10.42	3.35	9.62	1	0.002			
	RHI score	− 2.006	0.85	5.54	1	**0.019**	0.13	0.02	0.714
	HIF1A	0.12	0.05	5.08	1	**0.024**	1.13	1.017	1.267
	VEGF	0.01	0.00	6.96	1	**0.008**	1.01	1.004	1.030
	GREM-1	3.56	1.38	6.63	1	**0.010**	35.17	2.342	528.178
	VEGFC 2578ACC	− 0.37	0.82	0.20	1	0.653	0.69	0.137	3.474

a. The reference category is: 0 (healthy controls)

b. The reference category is: 1,0 (diabetics without DFS)

1.0: diabetic subjects without DFS

2.0 diabetic subjects with DFS

2,0 diabetic subjects with DFS

VEGF: Vascular Endothelial Growth Factor; NOS: endothelial Nitric-Oxid Synthase; HIF-1 alfa: *Hypoxia-inducible factor 1*-*alpha; GREM-1: Gremlin 1; RHI: reactive hyperaemia index*

## Discussion

In our study, we assessed the presence of epigenetic modifications and their effects on SNPs and miRNAs by correlating them with markers of adipo-inflammation and endothelial dysfunction in a diabetic population with and without lower limb ulcerative complications.

To our knowledge, few studies have evaluated surrogate indices of cardiovascular diseases, such as endothelial function indices and serum levels of inflammatory adipokines, in a diabetic population with lower limb ulcers [3].

To the best of our knowledge, there are no studies in the literature that have correlated the effects of epigenetic changes on single-nucleotide polymorphisms of genes encoding inflammatory molecules, such as IL-6 and TNF-alpha, and proangiogenic molecules, such as eNOS, VEGF, and HIF-1alpha, with endothelial dysfunction, noninvasively assessed by RHI; with serum levels of inflammatory molecules implicated in these pathways; or with new inflammatory adipokines, such as Gremlin-1, in a population of people with diabetes with and without lower limb ulcers.

We found that patients with diabetic foot syndrome showed a significant reduction in RHI values and a greater degree of endothelial dysfunction than diabetic controls without foot ulcers and healthy controls.

Peripheral arteriopathy is one of the most critical risk factors for diabetic foot, and the contribution of peripheral arteriopathy, together with infection and neuropathy, appears to be crucial.

Siasos et al. [26] provided the first evidence that diabetic foot in type 2 diabetes mellitus is associated with endothelial dysfunction. However, only one other study [4] examined endothelial function via RH-PAT, so our results appear to be important in validating this correlation. In addition, although the most common technique used to measure endothelial dysfunction is flow-mediated vasodilation, RH-PAT is a nonoperator-dependent and noninvasive solution, and the equipment is significantly less expensive.

The RH-PAT device records endothelium-mediated changes in the digital sphygmic wave, known as the PAT (peripheral arterial tone) signal, measured with a pair of state-of-the-art plethysmographic probes placed on the index fingers of both hands.

Endothelium-mediated changes in the PAT signal are elicited by creating a downstream hyperaemic reaction, so even the earliest stages of vascular pathology can be identified by measuring endothelial function. The most significant consequence of endothelial dysfunction is the initiation of an inflammatory process, which leads to the formation of atherosclerosis and its late sequelae. Moreover, endothelial dysfunction is involved in numerous systemic disease processes, such as erectile dysfunction, metabolic syndrome, cerebrovascular disease (TIA/stroke), preeclampsia, kidney failure, OSAS, PAD, and gangrene [27].

Normal endothelial function appears to be the key to cardiovascular health, as it plays a central role in mediating vessel tone and growth. Normal endothelial function also plays a crucial role in regulating the passage of circulating immune cells, as well as in the local regulation of haemostasis and coagulation. In contrast, the earliest alteration in most vascular pathologies is endothelial dysfunction. Furthermore, in arterial circulation, a healthy endothelium generally exerts a vasodilating influence on the vascular smooth muscle.

Diabetic foot is a micro- and macrovascular complication of diabetes. Endothelium-dependent vasodilation is markedly impaired in the coronaries of patients with hypertension, diabetes, ventricular hypertrophy, and other cardiovascular risk factors [28]. The loss of this vasodilation mechanism may contribute to the dysregulation of coronary flow. In addition, the earliest stages of epicardial atherosclerosis are associated with impaired endothelium-dependent coronary circulation dilatation, indicating that the pathophysiological consequences of atherosclerosis may extend to the human coronary microcirculation [21]. Thus, the micro- and macrovascular complications of diabetes are well represented by the evaluation of endothelium-dependent dilatation indices, such as the PAT index RHI.

Although the mean RHI values were not significantly different between diabetic patients with and without ulcerative complications of the lower limbs, there was an increased frequency of patients with RHI values < 1.60 in the DFS group compared with those in the diabetic and healthy control groups. Furthermore, these values are correlated with the diagnosis of endothelial dysfunction, so with equal mean RHI values, patients with diabetic foot are found to have a greater degree of endothelial dysfunction, as values of less than 1.67 RHI are indicative and predictive of endothelial dysfunction. These results appear to be in line with the results of our previous studies.

This finding could also help to explain further evidence from our study relating to poorer cognitive performance in patients with diabetic foot syndrome.

We showed that diabetic patients with or without diabetic foot showed significantly lower MMSE scores than healthy controls. Furthermore, although there was no significant difference in mean MMSE scores among the groups in the multiple regression analysis, a higher percentage of DFS patients, than those of controls with and without diabetes, had MMSE scores below 24, a value indicative of mild cognitive impairment and thus of early cognitive decline.

In a recent cross-sectional observational study [29], the 'ADELAHYDE' study, of patients with hypertension and memory disorders, an association of vascular changes (arterial hypertrophy and stiffness and endothelial dysfunction) with cognitive functions and with the presence of white matter hyperintensities (WMHs) on MRI was shown. These results confirmed the role of vascular factors in the evolution of cognitive function and in the onset of dementia.

Thus, in elderly hypertensive patients, vascular changes, independent of blood pressure levels, may play as important a role in memory disorders as white matter hyperintensities (WHMs). Therefore, arterial thickness and stiffness, as well as endothelial function, should be measured simultaneously and could represent an additional target for preventing memory disorders and WHMs.

In addition, another study conducted at our school [30] confirmed the presence of cognitive impairment, a greater degree of the severity of white matter lesions and a predisposition towards the development of hyperintense white matter lesions.

Further evidence from our study is the positive correlation between lower MMSE scores and hypertension and stroke occurrence. This positive correlation is in line with the findings of a previous study of our own group in which it was shown that patients with diabetic foot had a higher degree of cognitive decline, a higher number of white matter lesions, and a greater degree of severity of these lesions [31].This previous study found a negative correlation between white matter lesions and RHI values and a positive correlation between PWV and hyperintense white matter lesions in patients with diabetic foot syndrome. The authors corroborated that an MMSE score decline appeared to be linked to white matter lesions being positively correlated with PWV and negatively correlated with RHI values, which are considered surrogate markers of cardiovascular risk and hypertension [31].

This link would explain the positive correlation between stroke and hypertension found in our current study. One hypothesis for explaining the negative correlation between low MMSE scores and metformin use is that high glucose levels influence the occurrence of microvascular lesions in the cerebral circulation. Therefore, the use of metformin with its anti-glycaemic and metabolic properties for weight reduction could explain how altering these risk factors can reduce cerebral microvascular damage and cognitive decline [32].

In conclusion, MMSE scores of less than 24 and RHI values of less than 1.60 are closely associated with diabetic foot and, therefore, are factors predictive of this pathology. In clinical practice, the use of these tools could allow early diagnosis and thus greater control of risk factors to avoid the onset of ulcerative complications of the lower limbs.

We have also shown that patients with diabetic foot show significantly increased serum levels of Gremlin-1 compared to diabetic controls without ulcers and healthy people.

Gremlin-1 is an inflammatory adipokine belonging to the TGF-B family, which is highly produced by cells in adipose, muscle and liver tissue and can oppose the effects of insulin.

A recent study by McMahon et al. [33] demonstrated how Gremlin-1 expression is increased in mouse mesangial cells incubated with high levels of glucose/TGF-b or in renal cells in mice in which diabetic nephropathy was induced by bacterial infections. Furthermore, the same group demonstrated the overexpression of Gremlin-1 in patients with diabetic nephropathy three years later. Thus, these investigations introduced a new research scenario encouraging the analysis of Gremlin-1 overexpression in other pathological conditions, such as in patients with NAFLD on liver biopsy and in patients with type 2 diabetes mellitus and metabolic syndrome [34]. Therefore, Gremlin-1 could represent a novel serological marker of adipose inflammation.

Mitola et al. [35] identified the Gremlin-1 ligand VEGFR2. They emphasized how this adipokine, in addition to its role as an antagonist of the BMP pathway, was a genuine proangiogenic factor capable of binding to VEGF2 and triggering the signalling pathway precisely due to its ability to bind heparan sulfates.

Hedjazifar et al. further confirmed that Gremlin-1 was an inflammatory adipokine [34], and they observed that Gremlin-1 expression levels were higher in visceral adipose tissue, increased in obesity and further increased in patients with type 2 diabetes mellitus. In addition, the same authors discovered that Gremlin-1 possessed one last peculiar activity; it was able to oppose insulin-mediated intracellular signalling in human adipocytes, skeletal muscle, and hepatocytes.

To date, in no study has evaluated the role of Gremlin-1 in one of the micro- and macroangiopathic complications of diabetes, such as lower limb ulcers, been evaluated, which is why our results are innovative in this regard. As suggested by Mitola et al. [35], these increased levels could be explained by the evidence in our study of elevated serum VEGF levels in patients with diabetic foot syndrome. As already mentioned, Gremlin-1 appears to be a coactivator of VEGF and can induce microangiogenesis, a pathological process underlying the pathogenesis of diabetic ulcers. Moreover, a further hypothesis that may explain the increase in these serum levels may be that the cohort of patients with diabetic foot had higher BMI values, which are correlated with an excessive presence of adipose tissue and obesity, as demonstrated by Hedjazifar et al. [34].

Gremlin-1 represents the link that could clarify the dynamics between proangiogenic factors and endothelial remodelling. Mitola et al. [35] showed that Gremlin-1 is a VEGFR2 receptor agonist, but its peculiarity is that when present in monomeric form, it acts as a VEGF receptor antagonist [36, 37].

This evidence could further elucidate the cytovascular architecture in patients with endothelial damage, as the signalling pathway of vascular endothelial growth factor (VEGF) is altered, and the lack of expression of endothelial nitric oxide synthase (eNOS), whose activity and expression are stimulated by VEGF. Furthermore, it has recently been reported that bone morphogenesis proteins, such as Gremlin-1, may inhibit eNOS activity by binding to their respective receptor (BMPR2). These results are consistent with an autocrine action of Gremlin-1 in endothelial cells that mediates the hypoxia-induced reduction in eNOS expression and activity. This reduction in eNOS activity is functionally significant, as hypoxia-induced impairment of endothelial nitric oxide synthase activity contributes to vascular remodelling, the perpetuation of endothelial dysfunction and hypoxia-induced vasoconstriction. This evidence is supported by the work of Rowan et al. [38], who analysed the effects of hypoxia-related Gremlin-1 in the pulmonary microvascular endothelium.

Furthermore, this evidence explains certain results of our study, such as the significant increase in serum levels of VEGF and HIF-1alpha in subjects with diabetic foot and the absence of significantly increased serum levels of eNOS.

In regard to the molecular mechanisms of VEGF and eNOS in wound healing and ulcers in diabetic foot, we cannot overlook the role of HIF-1alpha. HIF-1alpha is an oxygen concentration-dependent transcription factor that can regulate multiple target genes and the expression of numerous proangiogenic growth factors, such as VEGF and eNOS. Thus, various hypoxia-induced adaptive reactions occur, such as angiogenesis, oxidative metabolism and cell apoptosis [39]. It has been reported that the healing disorder of diabetic ulcers is caused by poor angiogenesis, which leads to a failure to meet the metabolic demands of the granulation tissue [40]. Therefore, it is suggested that the promotion of local angiogenesis is likely to improve the healing process. In patients with diabetes with micro- and macrovascular complications, insufficient angiogenesis in wound healing may be caused by the expression of HIF1-alpha and its target molecules.

This finding suggests that Gremlin-1 can directly stimulate VEGF via the VEGFFr2 receptor, being a costimulator of VEGF, and activate, directly and indirectly, eNOS both via the VEGF pathway and by binding to BMP, respectively. Both mechanisms underlie the expression of HIF1-alpha, a factor required in the diabetic foot wound healing process, which, as already established on the basis of the findings of several studies [39, 40], is impaired in diabetic foot patients.

We have also shown how Gremlin-1 may be a predictive factor for diabetes and diabetic foot diagnosis. Furthermore, we have demonstrated how Gremlin-1 is also a predictive factor for diagnosing diabetic foot.

Thus, the concentration of circulating Gremlin-1 in patients with DFS could be a valuable biomarker of adipo-inflammation that can be used in predicting the diagnosis of diabetes and diabetic foot.

We also showed that patients with diabetic foot, compared to diabetic patients without lower limb ulcers and healthy controls, have higher serum levels of inflammatory molecules, such as HIF-1alpha and VEGF, and comparable levels of IL-6, TNF-alpha and eNOS.

HIF-1alpha, as an oxygen concentration-dependent transcription factor, can regulate several target genes, particularly VEGF. Thus, various hypoxia-induced adaptive reactions occur, such as angiogenesis, metabolism and cell apoptosis [41]. It has been reported that the healing disorder of diabetic ulcers is caused by poor angiogenesis, which leads to a failure to meet the metabolic demands of the granulation tissue [42], suggesting that the promotion of local angiogenesis is likely to improve the healing process. Our results are also in line with these previous studies.

VEGF is a molecule involved in various stages of diabetic ulcers. In particular, it is responsible for neoangiogenesis and granulation tissue formation.

Our study showed elevated serum levels of VEGF in diabetic patients with and without ulcerative complications of the lower limbs compared to those in healthy controls. This finding is in line with those of other studies, such as that of Zubair M et al. [43].

The increase in VEGF can be explained by the greater inflammatory background of the diabetic patient with a lower limb complication and the greater degree of endothelial dysfunction. Another explanation for the increased levels of VEGF is the elevated levels of Gremlin-1, which, being a coactivator of VEGF, also acts indirectly on both eNOS (reducing its values) and HIF-1alpha.

An elevated VEGF level was also found in patients with diabetic foot compared to those in controls with and without diabetes. In light of this result, an evaluation of the serum VEGF level as a serum inflammatory biomarker predictive of diabetic foot diagnosis may be advisable.

Although, in contrast to a previous study from our own group 83), no significant differences were found among the three groups regarding serum IL-6 and TNF-alpha levels, our results on serum markers of inflammation are in line with our previous work.

These results may represent the biological and pathological phenotype of an “adipo-vascular” axis, which involves both microvascular and inflammatory mechanisms. Furthermore, it may be further confirmed that in diabetic pathology, a crucial role is played by the metabolic and inflammatory microenvironment.

A possible explanation for the nonsignificant difference in serum levels of IL-6 and TNF-alpha among the three groups may be that these patients had been in the stressful condition of hospitalization for various diseases, which may have influenced their inflammation marker levels [44]. Furthermore, the percentage of patients who used antiplatelets and insulin was higher in diabetic patients with lower limb complications than in those without lower limb ulcers, and the percentage of patients who used ACE inhibitors and statins was higher in diabetic patients with lower limb complications than in healthy controls.

These drugs can act on the inflammatory background of atherosclerotic plaques and micro- and macrovascular damage. Furthermore, the percentage of patients who used metformin and GLP-1 analogues, drugs that act not only on glycaemic control but also on cardiovascular risks due to their anti-inflammatory action, was higher in diabetic patients without lower limb ulcers than in either patients with DFS or healthy controls.

Finally, within the healthy population, there were patients with hypertension and atherosclerosis, so this, together with the above explanations, could be another reason for the comparable serum IL-6 and TNF alpha levels observed among the three groups.

A further result of our study is the evidence of an increased presence of the VEGF C2578A gene polymorphism with predominant CC allele expression and reduced AC allele expression in patients with diabetic foot. These results are in line with the findings of the studies performed by Li X et al. [12] and Amoli et al. [13], who observed that the absence of the A allele might reduce susceptibility to a diabetic ulcer.

Furthermore, Bleda et al. [45] showed increased expression of VEGF C2578A CC in a population of patients with type 2 diabetes and peripheral arteriopathy.

In this sense, the demonstration of the presence of this polymorphism not only in the Chinese and Iranian populations but also in the Mediterranean population suggests that it may be a candidate marker for early diagnosis and a possible therapeutic target in patients with diabetic foot. Furthermore, this polymorphism appears to be predictive of a diagnosis of diabetes, and in this sense, testing for this polymorphism would allow early diagnosis of diabetes.

Our study did not find a significant frequency of the following polymorphisms: rs1800795 of the IL-6–174 gene, G > C, rs1800629 of the TNF-α-308 gene, G > A, rs11549465 of the HIF-1alpha-1772 gene, C > T and the ENOS-894 gene, G > T.

Viswanathan et al. [9], in a study on an Indian population in 2018, identified that the IL-6–174, G > C gene polymorphism was correlated with a higher risk of wound infection severity and worse ulcer staging. In addition, inflammatory cytokines had some influence on the same SNP. Similarly, Dhamodharan et al. [8], in a study on an Indian population with diabetic foot syndrome, and Erdogan et al. [10], in a study on Turkish people with diabetic ulcers, noted that this polymorphism conferred a certain degree of protection against diabetes but not against diabetic foot. Our results are, therefore, partly in line with the findings of Erdogan and Dhamodharan [8–10].

The study by Dhamodharan et al. [8] also showed that the SNP of the TNF-α-308 gene, G > A, conferred susceptibility to diabetes mellitus and diabetic neuropathy but not to diabetic foot. Thus, our results are in line with the findings in an Indian population.

Further study by Erdogan et al. [14] on a Turkish population with diabetic foot showed that the ENOS-894, G > T, and VEGF C936T polymorphisms were not risk factors for the onset of diabetic foot. The findings of this work are also in line with our results.

Pichu et al. [16], in a 2018 paper, identified that CT and TT allelic expression of the HIF-1alpha C772T gene polymorphism was increased in an Indian population with diabetic foot. However, a significant frequency of this polymorphism was not identified in our work. In contrast, we identified a high prevalence of CC, so our results differ from those of the abovementioned work, probably due to the ethnic difference between the populations under study.

In conclusion, concerning the analysis of genetic polymorphisms, our results show that patients with diabetic foot syndrome have increased expression of VEGF C2578A CC and reduced expression of VEGF C2578A AC. These results align with the works of Li X and Amoli [12, 13], highlighting a possible early and therapeutic marker role of this polymorphism.

In addition, the significant expression of the IL-6–174, G > C; TNF-α-308, G > A; HIF-1alpha-1772, C > T; and ENOS-894, G > T gene polymorphisms is partly in line with the findings of the studies performed by Erdogan, Pichu and Dhamodharan [8–16] and to a certain extent explicable by the ethnic differences in the investigated populations. However, further studies are needed to clarify the usefulness of testing for these polymorphisms as early markers of disease or therapeutic targets in patients with diabetic foot syndrome of various ethnic backgrounds.

Concerning the miRNA expression analysis, we identified that miR-217-5p and miR-503-5p were 2.19-fold and 6.21-fold more highly expressed in diabetic foot patients than in healthy controls, respectively. Moreover, diabetic patients without lower limb ulcer complications showed 2.41-fold and 2.24-fold higher miR-217-5p and miR-503-5p expression than healthy controls, respectively. However, no significant differences in the expression of these miRNAs were found when comparing diabetic patients with and without ulcerative lesions of the lower limbs.

Leeper NJ et al. showed that the overexpression of miRNA 503 appears to play a relevant role in the pathogenesis of diabetic foot. The increased expression of miRNA 503 leads to reduced angiogenesis and provokes a concomitant reduction in the re-epithelialization process. The two mechanisms act synchronously, impeding the healing process of diabetic ulcers [5].

The involvement of miRNA 217 has also been studied in the development process of diabetic ulcers. An in vivo study on mouse models showed that inhibiting miRNA 217 results in positive regulation of the HIF-1alpha pathway, promoting angiogenesis and inflammation and improving ulcer healing [7].

Our results concerning the significantly increased expression of miRNA 217 and miRNA 503 in patients with diabetic foot compared to healthy controls align with those reported in the literature. Furthermore, diabetic patients without ulcerative complications showed increased expression of these miRNAs compared to healthy subjects. The expression of these miRNAs may act on underlying molecular pathways, such as those of VEGF and HIF-1 alpha. These pathways also appear to play a relevant role in other minor vessel disease complications of diabetes, such as retinopathy and nephropathy, from which our diabetic patients were suffering. Therefore, this hypothesis could justify the nonsignificant difference in the expression of these miRNAs when comparing diabetic patients with and without ulcerative lesions of the lower limbs.

### Limitations

Because of the cross-sectional nature of the study, no possible conclusions on causality can be made. Nevertheless, regardless of when exposure and disease were ascertained relative to one another, a cross-sectional study may provide insights into the causal effects of exposure on disease incidence.

Another possible limitation is that the analysis of the candidate gene SNP data obtained in the present study may currently have some limitations in the study of complex disorders.

A further possible limitation is that although microRNAs are emerging as attractive therapeutic targets because of their small size, specific targetability, and critical role in disease pathogenesis, with < 20 miRNA target molecules having entered clinical trials, none have progressed to phase III trials. Thus, there is a possible limitation of this study due to the possible “translational” role of these miRNAs not being fully demonstrated.

## Conclusions

In patients with diabetic foot, we found predominant expression of the VEGF C2578A CC polymorphism and reduced expression of the AC allele.

Additionally, concerning the miRNA expression analysis, we found an overexpression of miR-217-5p and miR-503-5p in diabetic patients with and without diabetic foot syndrome.

We observed a significant increase in Gremlin-1 levels in the population of patients with diabetic foot, and we demonstrated how this inflammatory adipokine is a predictive marker for the diagnosis of diabetic foot. To date, no study has evaluated this inflammatory adipokine in diabetic foot, which, as already mentioned, makes our results innovative in the field.

Patients with diabetic foot syndrome had a higher degree of endothelial dysfunction and cognitive decline, as assessed by RH-PAT and the MMSE, respectively. This assessment of endothelial dysfunction in a noninvasive modality proved to be a fundamental early marker of cardiovascular risk in patients with diabetic foot syndrome. In fact, an RHI value of less than 1.6 was found to be predictive of the diagnosis of diabetic foot.

Finally, we showed that patients with diabetic foot syndrome have significantly increased serum levels of VEGF and HIF-1alpha.

In conclusion, in a population with diabetic foot, we demonstrated increased expression of the VEGF C2578A CC polymorphism; significantly elevated levels of serum markers of adipose inflammation, such as Gremlin-1, VEGF, and HIF; and an increased degree of endothelial dysfunction and cognitive decline. Many of these biomarkers are valuable and predictive in diagnosing diabetic foot syndrome. The detection of this single nucleotide polymorphism could allow early detection of the disease with stricter control of risk factors, and this SNP could also become a possible therapeutic target for gene therapy. However, further studies are needed to support and confirm our data and to identify new clinical and therapeutic scenarios in the study of diabetic foot.

## Supplementary Information

**Additional file 1:** STROBE Statement—checklist of items that should be included in reports of observational studies.

## Data Availability

All data and material are available on figshare. (https://figshare.com/account/items/23292947/edit).

## Abbreviations

NO: Nitric oxide
DFS: Diabetic foot syndrome
WHO: World Health Organization,
NSS: Neuropathy symptom score
ADA: American diabetes association
ESC: European society of cardiology
HDL: High-density lipoprotein
BMI: Body mass index
MMSE: Mini-mental state examination
RH-PAT: Reactive hyperaemia peripheral artery tonometry
RHI: Reactive hyperactivity index
HR: Heart rate
SVT: Supraventricular tachycardia (HR > 150 bpm)
VT: Ventricular tachycardia
SVBE: Supraventricular ectopic beat
EVB: Ventricular ectopic beat
TNF-α: Tumour necrosis factor alpha
IL-6: Interleukin 6
ACE: Angiotensin converting enzyme
GLP1-ra: GLP-1 receptor agonist
DPP-4 inh: Dipeptidyl peptidase-4 inhibitors
SBP: Systolic blood pressure
DBP: Diastolic blood pressure
BMI: Body mass index
RHI: Reactive hyperaemia index
MMSE: Mini-mental state examination
HbA1c: Glycated haemoglobin
TNF-alpha: Tumour necrosis factor-alpha
VEGF: Vascular endothelial growth factor
hsa-miR-217-5p: Human miRNA217-5p
hsa-miR-126-3p: Human miRNA126-3p
hsa-miR-503-5p: Human miRNA 503-5p
eNOS: Endothelial nitric-oxide synthase
HIF-1 alpha: Hypoxia-inducible factor 1-alpha
